# Bub1 Facilitates Virus Entry through Endocytosis in a Model of Drosophila Pathogenesis

**DOI:** 10.1128/JVI.00254-18

**Published:** 2018-08-29

**Authors:** Shuo Yang, Junjing Yu, Zhiqin Fan, Si-tang Gong, Hong Tang, Lei Pan

**Affiliations:** a CAS Key Laboratory of Molecular Virology and Immunology, Institut Pasteur of Shanghai, Chinese Academy of Sciences, Shanghai, China; b CAS Key Laboratory of Infection and Immunity (CASKLII), Institute of Biophysics, Beijing, China; c The Joint Center for Infection and Immunity, Guangzhou Institute of Pediatrics, Guangzhou Women and Children's Medical Center, Guangzhou, China; d College of Life Sciences, University of Chinese Academy of Sciences, Beijing, China; University of Southern California

**Keywords:** Drosophila, *bub1*, endocytosis, virus entry

## Abstract

In order to establish productive infection and dissemination, viruses usually evolve a number of strategies to hijack and/or subvert the host defense systems. However, host factors utilized by the virus to facilitate infection remain poorly characterized. In this work, we found that Drosophila melanogaster deficient in *budding uninhibited by benzimidazoles 1* (*bub1*), a highly conserved subunit of the kinetochore complex regulating chromosome congression (1), became resistant to Drosophila C virus (DCV) infection, evidenced in increased survival rates and reduced viral loads, compared to the wild-type control. Mechanistic analysis further showed that Bub1 also functioned in the cytoplasm and was essentially involved in clathrin-dependent endocytosis of DCV and other pathogens, thus limiting pathogen entry. DCV infection potentially had strengthened the interaction between Bub1 and the clathrin adaptor on the cell membrane. Furthermore, the conserved function of Bub1 was also verified in a mammalian cell line. Thus, our data demonstrated a previously unknown function of Bub1 that could be hijacked by pathogens to facilitate their infection and spread.

**IMPORTANCE** In this work, we identify for the first time that the nuclear protein Bub1 (budding uninhibited by benzimidazoles 1), a highly conserved subunit of the kinetochore complex regulating chromosome congression, has a novel and important function on the cell membrane to facilitate the virus to enter host cells. Bub1 deficiency empowers the host to have the ability to resist viral infection in Drosophila and a human cell line. Bub1 is involved in the virus entry step through regulating endocytosis. The DCV capsid protein can recruit Bub1, and DCV infection can strengthen the interaction between Bub1 and a clathrin-dependent endocytosis component. The restricted entry of vesicular stomatitis virus (VSV) and Listeria monocytogenes in *bub1*-deficient flies and cell lines was also observed. Therefore, our data implicate a previously unknown function of Bub1 that can be hijacked by pathogens to facilitate their entry, and Bub1 may serve as a potential antiviral therapy target for limiting viral entry.

## Abstract (precis)

In this work, we identify for the first time that the nuclear protein Bub1 (budding uninhibited by benzimidazoles 1), a highly conserved subunit of the kinetochore complex regulating chromosome congression, has a novel and important function on the cell membrane to facilitate the virus to enter host cells. Bub1 deficiency empowers the host to have the ability to resist viral infection in Drosophila and a human cell line. Bub1 is involved in the virus entry step through regulating endocytosis. The DCV capsid protein can recruit Bub1, and DCV infection can strengthen the interaction between Bub1 and a clathrin-dependent endocytosis component. The restricted entry of vesicular stomatitis virus (VSV) and Listeria monocytogenes in *bub1*-deficient flies and cell lines was also observed. Therefore, our data implicate a previously unknown function of Bub1 that can be hijacked by pathogens to facilitate their entry, and Bub1 may serve as a potential antiviral therapy target for limiting viral entry.

## INTRODUCTION

Virus-host coevolution offers a mountain of knowledge of the interaction between viruses and hosts. The host evolves many antiviral mechanisms to recognize and defend against invasion by viruses (2, 3), while viruses coevolve to utilize host cell machinery for their binding, entry, replication, and shedding (4). Thus, to achieve a head start in this “arms race,” the identification of host factors that can be hijacked by viruses becomes increasingly important for a better understanding of virology and pathology in human viral diseases.

Drosophila melanogaster has been proven to be a powerful and productive system to investigate host-virus interactions *in vivo* (5, 6) because of its highly conserved antiviral innate immune signaling pathways (7–10). Four well-established major cytosolic antiviral pathways in Drosophila, including the RNA interference (RNAi) pathway, the JAK-STAT pathway, the NF-κB pathway, and the autophagy pathway (11, 12), target different steps of the viral life cycle and have significant implications for human antiviral studies. The RNAi mechanism provides a broad spectrum of antiviral activities in the blockage of viral genome transcription (13, 14), while the inducible JAK-STAT signaling pathway has been found to offer efficient defense specifically against viruses of the Dicistroviridae family (e.g., Drosophila C virus [DCV] and Cricket paralysis virus [CrPV]) (8). Two NF-κB pathways in Drosophila, Toll and immune deficiency (IMD), have been reported to not only play major roles in antibacterial activities but also function in response to viral infections (15–17). Additionally, the autophagy pathway contributes to antiviral potency to limit Vesicular stomatitis virus (VSV) and Rift Valley fever virus (RVFV) infection in flies (9, 18, 19).

DCV, a single-positive-stranded RNA virus, is well studied and broadly used in the Drosophila screening system (20), due to its high infection-caused mortality rate in wild-type flies (21). To identify potential host factors hijacked by the virus, we set up a pilot genetic screen for mutant genes that can enable mutant flies to resist DCV infection. We found that a mutant of *CG14030*, which harbored the Drosophila *bub1* gene (orthologous to human *bub1*) (1), was resistant to DCV-induced mortality. Bub1 was previously reported to play a key role in the establishment of the mitotic spindle checkpoint and chromosome congression by forming a complex with BubR1 and Bub3 in binding to kinetochores (22). Recently, one study suggested that Bub1 also interacts with and stabilizes the TGFβ receptor I/II (TGFβRI/II) complex to enhance transforming growth factor β (TGFβ) signaling in the cytoplasm (23). In this study, we found a novel function of Bub1, in that *bub1* deficiency could limit virus entry, possibly through interfering with clathrin-mediated endocytosis of viruses and other pathogens.

## RESULTS

### Bub1-deficient flies are more resistant to DCV infection.

**FIG 1:**
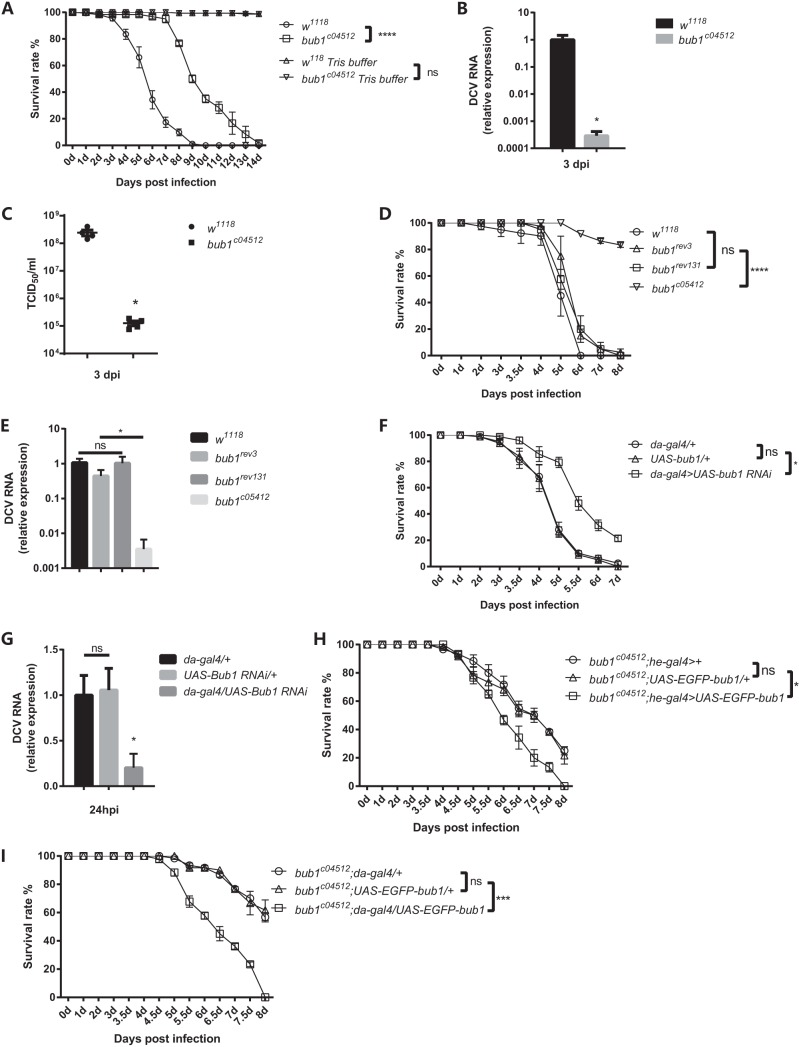
*bub1*-deficient flies become resistant to DCV infection. (A) Survival rates of Wolbachia-free *bub1*-deficient flies and wild-type (*w^1118^*) flies after DCV injection. (B) DCV RNA levels in the whole body of the indicated flies were measured by qRT-PCR at the indicated times and normalized to that in *w^1118^* flies. (C) DCV titers from the whole body of the indicated flies calculated by CPE at the indicated times. TCID_50_, 50% tissue culture infective dose. (D) Survival rates of *w^1118^* flies and *bub1^c04512^* mutant flies with a precise pBac element deletion (*bub1^rev3^* or *bub^rev131^*). (E) DCV RNA levels in the whole body of the indicated flies were measured by qRT-PCR at 3 days postinfection (dpi) and normalized to that in *w^1118^* flies. (F) Survival rates of files with ubiquitous knockdown of *bub1* and the corresponding genetic control flies after DCV injection. (G) DCV RNA levels from the whole body of the indicated flies were measured by qRT-PCR at the indicated times and normalized to that in *da-gal4>+* flies. (H) Survival rates of *bub1^c04512^* flies with reintroduced *bub1* in hemocytes and corresponding genetic controls after DCV infection. (I) Survival rates of *bub1^c04512^* flies with reintroduced *bub1* in whole body and corresponding genetic controls after DCV infection. All error bars represent SE of data from at least three independent tests (*n* > 60 flies [A, D, F, H, and I] for each line or *n* > 15 [B, C, E, and G] for each time point). *, *P* < 0.05; ***, *P* < 0.001; ****, *P* < 0.0001; ns, not significant (as determined by a Kaplan-Meier test [A, D, F, H, and I], Student's *t* test [B and C], or two-way ANOVA [E and G]).

To identify potential host factors participating in antiviral responses, we developed a machine-learning algorithm using a support vector machine to score each Drosophila gene according to the likelihood of involvement in viral infection (our unpublished data). Subsequently, approximately 110 top-scoring genes were set as the candidates in a genetic screen for an abnormal innate response to DCV infection. Around 60 viable homozygous/heterozygous mutant lines or RNAi lines, particularly the “hit” genes with mammalian orthologues, were further phenotypically validated repeatedly (see Fig. S1A in the supplemental material). Mutation of the gene *CG14030* harboring Drosophila *bub1* seemed to give flies strong resistance to DCV infection (Fig. S1A). To investigate the role of Bub1 in viral infection in Drosophila, homozygous mutant flies (*bub1^c04512^*) induced by the insertion of a transposable piggyBac element in the 3′ untranslated region (UTR) of the *bub1* gene were applied for nanoinjection of DCV (Fig. S2A). Of note, symbiotic Wolbachia bacteria were reported to increase resistance to RNA virus infection in Drosophila melanogaster (24). To exclude the possibility that the difference of Wolbachia densities in flies might affect susceptibility to DCV, flies used in this study were Wolbachia free. After DCV injection, *bub1^c04512^* mutant flies survived DCV infection much better than the genetic wild-type control *w^1118^* flies, the latter of which presented consistently increased mortality rates (Fig. 1A and Fig. S2B). Both quantitative real-time PCR (qRT-PCR) of DCV RNA levels (Fig. 1B) and cytopathic effect (CPE) assays (Fig. 1C) showed that DCV loads in *bub1^c04512^* flies were significantly lower than those in *w^1118^* flies after viral infection. Therefore, flies with *bub1* deficiency became resistant to DCV infection, likely due to the reduction of pathogen loads.

The presence of the S or R allele of the *pastrel* (*pst*) gene in the genetic background was reported to affect DCV infection (25, 26). To further confirm whether Bub1 was necessary for virus infection, precise excision of the inserted piggyBac element by the pBac transposase was performed in *bub1^c04512^* flies, which restored *bub1* expression to the level in wild-type flies (Fig. S2A). Moreover, both yielded flies (*bub1^rev3^* and *bub1^rev131^*) and *bub1^c04512^* flies shared the same genetic background containing the R allele of the *pst* gene (Fig. S2C). Although wild-type *w^1118^* flies had the S allele of the *pst* gene, the survival rates of recovered *bub1^rev3^* and *bub1^rev131^* flies were still comparable to those of *w^1118^* flies but much lower than those of *bub1^c04512^* flies after DCV infection (Fig. 1D), and there were much higher DCV loads in recovered *bub1^rev3^* and *bub1^rev131^* flies than in *bub1^c04512^* mutant flies (Fig. 1E). These results indicated that the resistance of *bub1^c04512^* flies to DCV infection is probably attributed to the *bub1* mutation rather than different *pst* genotypes. Furthermore, flies (*da-gal4/+*;*UAS-bub1-RNAi/+*) with ubiquitous knockdown of *bub1* using the upstream activation sequence (UAS)-Gal4 system (27) (Fig. S2D) showed significantly reduced mortality rates (Fig. 1F) and low DCV loads (Fig. 1G), even at 24 h postinfection (hpi), compared to control flies. Therefore, *bub1* is required for DCV infection and enhances the mortality and morbidity of flies after infection.

Hemocytes participate in the humoral immune response against virus infection in Drosophila (19, 28, 29). To determine whether *bub1* in hemocytes had any role in DCV infection, we reintroduced the *bub1* gene specifically in hemocytes of *bub1^c04512^* flies by *he-gal4* (Fig. 1H). This complementation increased the susceptibility of flies to DCV infection, similar to flies with the whole-body complementation of *bub1* by *da-gal4* (Fig. 1I). Therefore, this suggests that *bub1* in hemocytes plays an important role in the response to DCV infection.

### DCV tolerance of Bub1-deficient flies is not dependent on enhanced canonical antiviral signaling activity.

**FIG 2:**
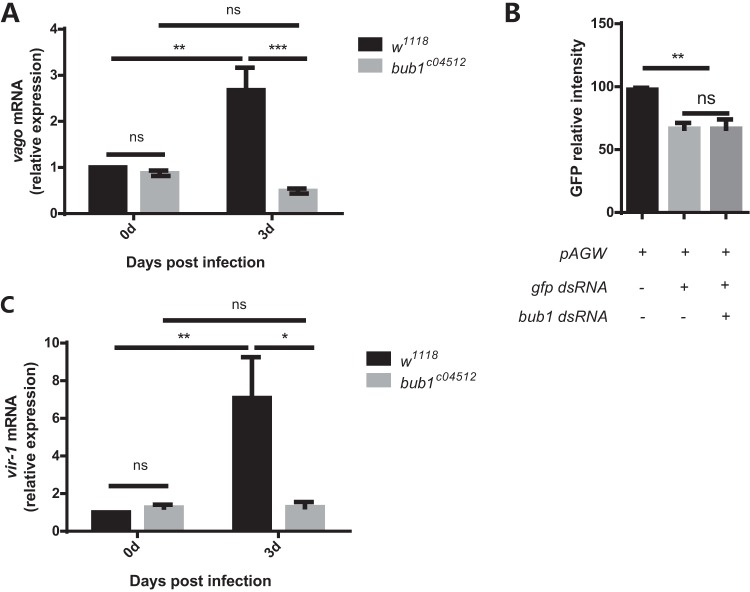
*bub1* deficiency does not show enhanced Dcr2/RNAi and JAK-STAT antiviral signaling activity. (A and C) *vago* (A) and *vir-1* (C) mRNAs expressed in the whole body, measured by qRT-PCR at 3 days postinfection. The change of expression was normalized to that of *w^1118^* flies at day 0. (B) GFP fluorescence intensity in S2* cells cotransfected with pAGW and *gfp* dsRNA in the presence or absence of *bub1* dsRNA. All data were normalized to those for S2* cells transfected with only pAGW. All error bars represent SE of data from at least three independent tests (*n* > 15 flies [A and C] for each time point). *, *P* < 0.05; **, *P* < 0.01; ***, *P* < 0.001; ns, not significant (as determined by two-way ANOVA [A and C] or one-way ANOVA [B]).

DCV infection in Drosophila can activate several typical antiviral signaling pathways, such as the Dicer-2/RNAi pathway and the JAK-STAT pathway. The NF-κB and autophagy pathways were reported not to be involved in the anti-DCV response in Drosophila (9, 17, 30). To substantiate whether *bub1* mutation evoked the known cytosolic antiviral mechanisms, additional experiments were performed. Activation of Dicer-2/RNAi signaling can increase the expression level of *vago* (7). However, *vago* transcription was upregulated in *w^1118^* flies but still remained at basal levels in *bub1* mutant flies after DCV infection (Fig. 2A). Furthermore, Bub1 knockdown in S2* cells did not affect double-stranded RNA (dsRNA)-mediated *gfp* gene silencing in RNAi efficiency assays (Fig. 2B). Therefore, the loss of *bub1* could not elevate Dicer-2/RNAi signaling activity after DCV infection.

JAK-STAT signaling is another important anti-DCV pathway in Drosophila. *vir-1* is a widely used reporter gene of the JAK-STAT pathway in response to DCV infection (8). Intriguingly, *vir-1* expression in *bub1^c04512^* flies was barely upregulated and significantly lower than that in *w^1118^* flies after DCV infection (Fig. 2C). These results suggest that the low activity of JAK-STAT signaling in *bub1* mutant flies could not be the reason for viral resistance.

### Bub1 participates in viral entry.

**FIG 3:**
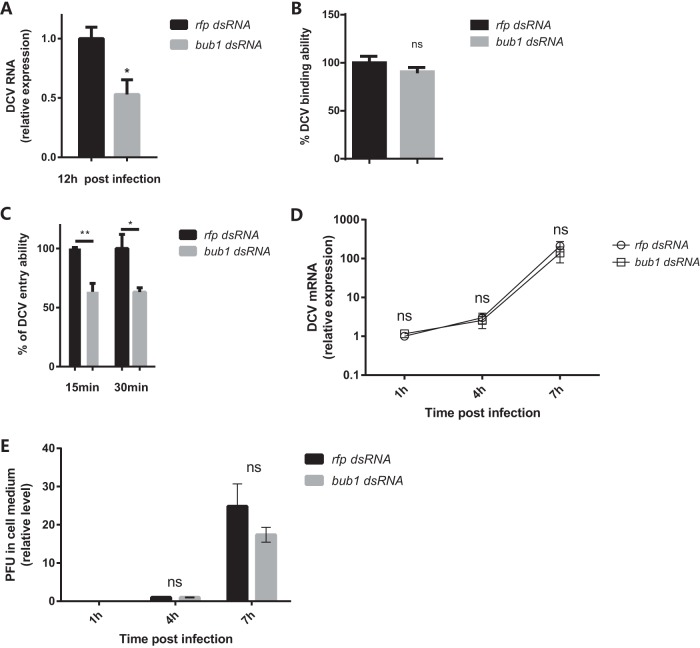
*bub1* deficiency reduces DCV entry. (A) Decreased virus replication when Bub1 is silenced. DCV RNA levels in the indicated cells were measured by qRT-PCR at the indicated times and normalized to that in *rfp* dsRNA-treated cells. (B) *bub1* knockdown in S2* cells does not affect DCV binding ability. The DCV binding ability was indicated by the DCV genome RNA level quantified by qRT-PCR and normalized to the value for the control group treated with *rfp* dsRNA. (C) DCV entry ability in S2* cells with or without *bub1* knockdown at 30 min postinfection. The DCV genome RNA level was quantified by qRT-PCR. The percent DCV entry ability was calculated by the equation *y/x̄* × 100, where*x̄* indicates the mean value for DCV RNA from the *rfp* dsRNA group and *y* indicates the values for DCV RNA from the *rfp* dsRNA group or *bub1* dsRNA group. (D) *bub1* knockdown in S2* cells does not affect virus replication. DCV RNA levels from S2* cells treated with *rfp* dsRNA or *bub1* dsRNA were normalized to the value for each self-group at 1 hpi. (E) *bub1* knockdown in S2* cells does not affect virus release. The relative PFU level in the supernatant was normalized to the value for each self-group at 4 hpi. All error bars represent SE of data from at least three independent tests. *, *P* < 0.05; **, *P* < 0.01; ns, not significant (as determined by two-way ANOVA [D and E] or Student's *t* test [A to C]).

The lack of strengthened antiviral signaling activities in *bub1* mutant flies prompted us to investigate whether the viral life cycle could be compromised. Similar to *in vivo* experiments in flies, knockdown of *bub1* in Drosophila S2* cells by dsRNA likewise led to decreased virus loads after DCV infection (Fig. 3A; see also Fig. S3A in the supplemental material for knockdown efficiencies). Of note, previous studies mentioned that only Bub1 depletion did not induce a significant influence on the proliferation and viability of human cells (31, 32). Indeed, partial knockdown of *bub1* in S2* cells had no effects on cell proliferation and viability as well, at least in our very short experimental time frame (Fig. S3B and S3C). Hence, the viral life cycle, including binding, entry, replication, and release, was tested in S2* cells. Clathrin-mediated endocytic entry is one of the rate-limiting steps for DCV infection, which can be blocked at 4°C without affecting DCV binding (6). Knockdown of *bub1* by dsRNA did not influence the efficiency DCV attachment to S2* cells (4°C at a multiplicity of infection [MOI] of 100) (Fig. 3B; see also Fig. S3D in the supplemental material for the DCV binding kinetic curve, which was in line with linear regression from an MOI of 6.25 to an MOI of 100). After viral entry was released at 28°C for 15 or 30 min (see Fig. S3E in the supplemental material for DCV entry kinetics, which was in line with linear regression within 30 min), much less DCV virus was absorbed by S2* cells with *bub1* knockdown than the nonspecific dsRNA controls (Fig. 3C). Critically, *bub1* deficiency did not affect virus genome replication (Fig. 3D) or virion release (Fig. 3E) in S2* cells. Therefore, these results indicate that *bub1* deficiency reduced viral loads at the level of cell entry.

### Bub1 enhances viral entry through endocytosis.

**FIG 4:**
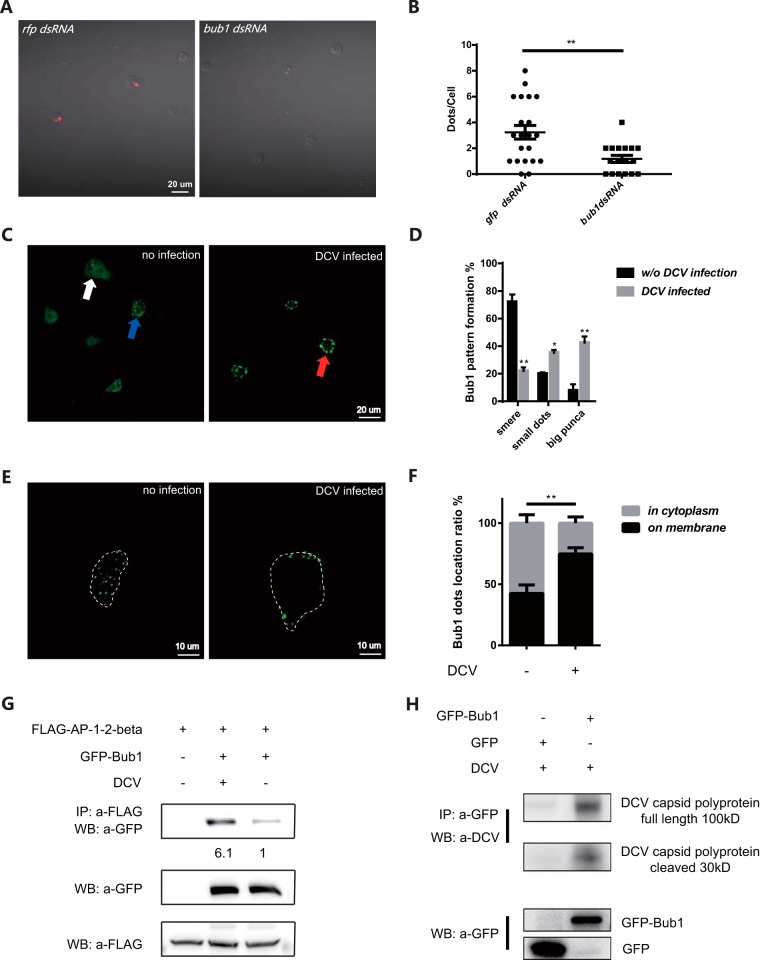
Bub1 is involved in regulation of endocytosis. (A) *bub1* knockdown impairs intake of fluorescence-labeled dextran in S2* cells. Dextran intake was observed by using a Zeiss LSM700 confocal microscope. (B) *bub1* knockdown in S2* cells significantly reduces endocytosis ability, which was quantified by calculating the number of fluorescent dextran dots in cells. (C) Location of Bub1-GFP in S2* cells with or without DCV infection at 1 hpi at an MOI of 100. All images were taken by using a Zeiss LSM700 confocal microscope. White arrow, smeared pattern formation; blue arrow, small dots (diameter of <1 μm); red arrow, large puncta (diameter of ≤1 μm). (D) Statistics for the indicated Bub1 location pattern before (*n* = 215) and after (*n* = 220) DCV infection. (E) Bub1 protein forms large puncta on the cell membrane after DCV infection. Cell membranes are indicated by a white dashed line according to the bright-field channel. (F) The ratio of Bub1 protein that formed dots in the cytoplasm or on the cell membrane with (*n* = 256)/without (*n* = 230) DCV infection. (G) DCV infection strengthens the Bub1/AP1-2-beta interaction. Transfected cells were infected with DCV at an MOI of 100 for 1 h. The relative intensity of the GFP-Bub1 band in immunoprecipitation (IP) normalized to the FLAG-AP1-2-beta input is indicated. WB, Western blot. (H) Bub1 interacts with DCV capsid polyprotein. Transfected cells were infected with DCV at an MOI of 100 for 1 h. All error bars represent SE of data from at least three independent tests. *, *P* < 0.05; **, *P* < 0.01 (as determined by Student's *t* test [B, D, and F]). Three repetitions were performed.

Next, we set up a dextran intake assay to determine whether Bub1 was involved in the regulation of endocytosis, the major pathway of DCV entry. *bub1* knockdown led to significantly less uptake of fluorescence-labeled dextran in S2* cells (Fig. 4A and B), indicating reduced endocytic activity. Previously, a yeast two-hybrid experiment suggested that Bub1 might interact with members of the adaptin superfamily, which mediate the formation of clathrin-coated pits (33, 34), and heterozygous flies with a partial loss of function of *ap-1u* or *syt7*, the components of the clathrin-dependent endocytosis complex (35, 36), could phenocopy *bub1* mutant flies in resistance to DCV infection (see Fig. S4A in the supplemental material). Thus, these results further suggest that Bub1 might be required in the regulation of clathrin-mediated endocytosis.

However, *bub1* expression remained steady before and after DCV infection *in vivo* (Fig. S4B). Simply overexpressing *bub1* in S2* cells did not increase the ability for viral entry (Fig. S4C) or viral loads (Fig. S4D). These results prompted us to investigate the localization of Bub1 in response to DCV infection. Fluorescence microscopic analysis showed that Bub1 remained in a smear cytoplasmic distribution in 72% of S2* cells wherein the green fluorescent protein (GFP)-Bub1 fusion protein was overexpressed. Interestingly, DCV stimulation promoted the Bub1 protein to form large puncta in 43% of S2* cells, compared with only 8% before infection (Fig. 4C and D). Closer inspection showed a significant increase of punctum recruitment to the cell membrane after DCV infection, compared to that before infection (65% versus 43%) (Fig. 4E and F). More importantly, coimmunoprecipitation experiments showed that DCV infection caused an increase in the interaction between Bub1 and β-adaptin (Fig. 4G); the latter is the core component that mediates the formation of vesicles by clathrin-coated pits (34). In line with a previous study showing that Bub1 had the ability to interact with simian virus 40 (SV-40) (37, 38), the Bub1-GFP fusion protein preferred to bind to the DCV capsid protein rather than the GFP control in coimmunoprecipitation assays (Fig. 4H). Together, these results support the idea that more Bub1 might be recruited on the cell membrane by DCV infection to assist virus entry through clathrin-dependent endocytosis.

### Bub1 has a conserved function to facilitate endocytosis-dependent pathogenic infection.

**FIG 5:**
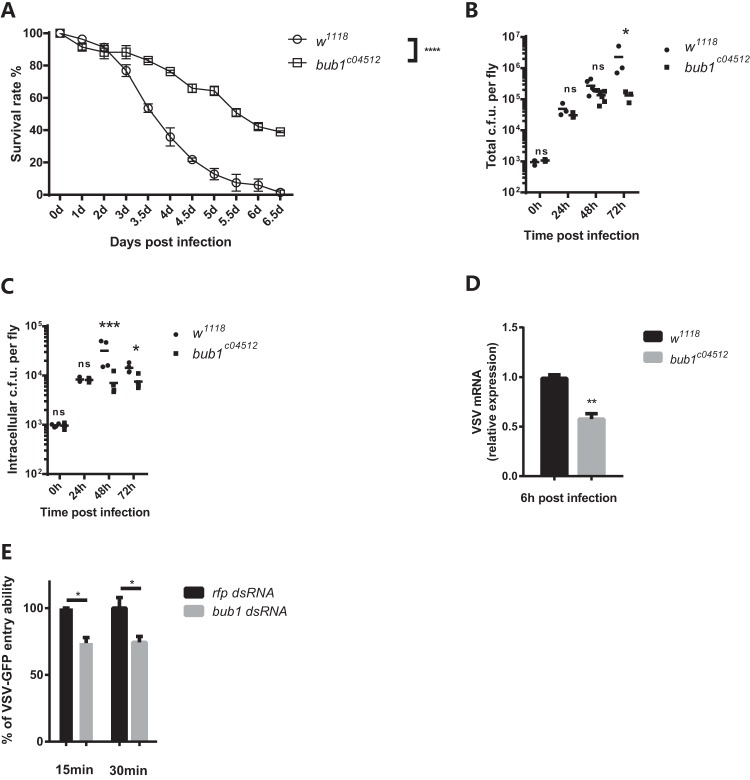
Bub1 facilitates endocytosis-dependent pathogen infection. (A) *bub1*-deficient flies are resistant to Listeria monocytogenes septic infection compared to wild-type (*w^1118^*) flies. (B) Total CFU of Listeria monocytogenes from the whole body of the indicated flies at the indicated times. (C) Intracellular CFU of Listeria monocytogenes from the whole body of the indicated flies at the indicated times. Gentamicin was injected into the fly anterior abdomen 3 h before sample collection to eliminate extracellular bacteria. (D) VSV RNA levels in the whole body of the indicated flies were measured by qRT-PCR at the indicated times and normalized to that in *w^1118^* flies. (E) *bub1* knockdown in S2* cells impairs VSV-GFP entry ability at 15 min and 30 min postinfection. VSV RNA was quantified by qRT-PCR. The percent VSV entry ability was calculated as mentioned above. All error bars represent SE of data from at least three independent tests (*n* > 60 flies [A] for each line or *n* > 20 flies [B to D] for each time point). *, *P* < 0.05; **, *P* < 0.01; ***, *P* < 0.001; ****, *P* < 0.0001; ns, not significant (as determined by a Kaplan-Meier test [A], Student's *t* test [D and E], or two-way ANOVA [B and C]).

**FIG 6:**
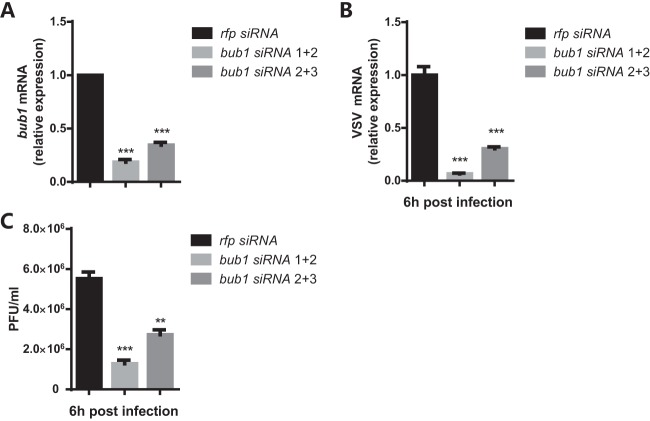
Bub1 has conserved function in mammalian cells. (A) *bub1* knockdown efficiency in 293T cells, measured by qRT-PCR. The change of expression was normalized to that in *rfp* siRNA-treated cells. (B) VSV-G mRNA levels in the indicated cells measured by qRT-PCR at the indicated times. The change of expression was normalized to that in *rfp* siRNA-treated cells. (C) VSV titers in the indicated cells measured by a plaque assay at the indicated times. All error bars represent SE of data from at least three independent tests. **, *P* < 0.01; ***, *P* < 0.001 (as determined by two-way ANOVA [A to C]).

Since Bub1 associated with common endocytic machinery (Fig. 4A and B), we then set out to address whether Bub1 was involved in the endocytosis of other pathogens. Both Listeria monocytogenes and Vesicular stomatitis virus (VSV) enter cells via endocytosis (39, 40). Similar to DCV infection, *bub1^c04512^* flies became more resistant to L. monocytogenes infection (Fig. 5A), with much lower bacterial loads (Fig. 5B), than *w^1118^* controls. The decrease in intracellular numbers of L. monocytogenes bacteria was further indicative of a defect in cell entry (Fig. 5C), as gentamicin was used to specifically kill extracellular bacteria (41). VSV can establish a noncytopathic persistent infection in Drosophila melanogaster cells (42, 43) and does not induce fly death under normal conditions (44). Thus, there was no difference in survival rates between *bub1^c04512^* and *w^1118^* flies (see Fig. S5A in the supplemental material). However, *bub1^c04512^* flies present significant lower VSV loads than *w^1118^* control flies (Fig. 5D). Similar to DCV infection, knockdown of *bub1* reduced VSV entry in S2* cells as well (Fig. 5E). Furthermore, knockdown of human *bub1* (Fig. 6A) also led to reduced VSV RNA levels (Fig. 6B) and titers (Fig. 6C) in 293T cells. These results suggest that Bub1 might serve as a conserved fundamental endocytic factor that facilitates cell entry of pathogens and, hence, microbial pathogenesis.

## DISCUSSION

Viruses are some of the major infectious agents that can provoke epidemic diseases. Thus, a better understanding of the interaction between viruses and the host will provide valuable insights into prevention and treatment of viral infection. Aside from deciphering antivirus signaling and regulators, host factors that can be utilized by the virus to facilitate infection need to be fully characterized. In this study, a DCV infectious model was established in Drosophila, and a pilot genetic screen for mutants resistant to virus infection was performed. To our surprise, the *bub1* gene was identified to enhance flies' mortality following DCV infection, as the preponderance of literature describes Bub1 as mainly functioning in the nucleus as a component of the mitotic machinery to regulate chromatid segregation (45, 46). This finding also reflects the fact that virus coevolves in a very smart way to target such a conservative and critical machinery protein of the host. Moreover, we present evidence here that Bub1 could be hijacked on the cell membrane to help the pathogen enter the host through regulating endocytosis.

Drosophila flies with *bub1* deficiency resisted DCV infection, accompanied by a very low virus titer. These results prompted us to investigate whether Bub1 played roles in antiviral signaling pathways. Unexpectedly, either the Dcr2/RNAi pathway or the JAK-STAT pathway, the major anti-DCV pathways in Drosophila, exhibited reduced rather than enhanced activity in *bub1* mutant flies after DCV infection. This was probably due to the much lower virus loads in *bub1* mutant flies, which hinted at the fact that *bub1* deficiency could limit virus amounts in hosts prior to the initiation of antiviral signaling. Indeed, further detailed analysis showed that a loss of function of Bub1 markedly reduced the entry ability of DCV through regulating endocytosis. However, not limited to DCV, deficiency of *bub1* also protected flies from infection by other endocytosis-dependent pathogens, such as VSV and Listeria.

The transcriptional levels of *bub1* did not show any statistical differences before and after viral infection. Interestingly, coimmunoprecipitation assays indicated that the DCV capsid protein might interact with Bub1, and virus infection promoted more Bub1 protein to translocate onto the cell membrane and gather as large puncta. This suggests that Bub1 might be hijacked by DCV and function as a mediator to orchestrate endocytic components. However, whether other endocytosis-dependent pathogens, like DCV, recruit Bub1 directly or indirectly through endocytic pits is well worth testing. Furthermore, spatiotemporal colocalization between Bub1 and endocytic complex components along with pathogen infection also need deep analysis. However, our results showed that DCV infection strengthened the interaction between Bub1 and β-adaptin. This warranted further investigation into whether Bub1 could potentiate and stabilize endocytic complex formation in response to viral infection. Previous studies have described that Bub1 locates in the nucleus and acts as a key factor in establishing the mitotic spindle checkpoint with binding to kinetochores (45, 46). Here, we found that Bub1 has a novel function in the cytoplasm to assist virus infection by regulating endocytosis. However, how the virus utilizes and initiates Bub1 function in the cytosol needs better analysis and further investigation.

It should be noted that a recent study found that Bub1 can function as a scaffold protein to stabilize TGFβRI/II complex formation on cell membrane, dependent on its Ser/Thr kinase activity, to promote TGFβ signaling (23). The TGFβ signaling pathway has been well studied in the regulation of innate and adaptive immune responses (47). Some of the literature suggested that TGF signaling can play immunosuppressive roles to promote virus replication and pathogenesis (48–50). However, blocking Bub1 kinase activity by high levels of 2-[(4-amino-1-(*tert*-butyl)-1*H*-pyrazolo[3,4-*d*]pyrimidin-3-yl)methyl]phenol (2OH-BNPP1) reduced DCV replication in S2* cells (see Fig. S6A in the supplemental material) but had no effects on the interaction between Bub1 and β-adaptin (Fig. S6B). Thus, it needs to be further determined whether reduced TGFβ signaling activity in *bub1*-deficient flies contributes to resistance to viral infection as well, through strengthening innate immune responses.

To this end, our work characterizes a host factor, Bub1, that has a previously unknown function to facilitate viral/microbial entry by regulating endocytosis activity. This discovery will promise to provide insights into avenues for therapeutic intervention, which may be applied to human infectious diseases.

## MATERIALS AND METHODS

### Fly stocks and culture.

The wild-type flies used were *w^1118^* (catalogue number 3605; Bloomington Stock Center). Unless specifically mentioned otherwise, all flies were maintained on standard cornmeal fly food (1 liter of food contains 77.7 g cornmeal, 32.19 g yeast, 10.6 g agar, 0.726 g CaCl_2_, 31.62 g sucrose, 63.2 g glucose, 2 g potassium sorbate, and 15 ml 5% Tegosept). Flies were cultured at room temperature under a normal light/dark cycle, unless noted otherwise. The Drosophila stocks used in this study are described in FlyBase (http://flybase.org/), unless specified otherwise. The mutant lines were *bub1^c04512^* (catalogue number BL11489; Bloomington Stock Center), *syt7^A426^* (catalogue number BL16111), and *ap-1*μ^*SHE-11*^ (catalogue number BL8190). The RNAi line was *UAS-bub1-RNAi* (catalogue number THU0133; Tsinghua Stock Center). The UAS line was *UAS-egfp-bub1* (Christian F. Lehner, University of Zurich).

To knock down *bub1* in the whole body, *da-gal4* virgins were crossed with *bub1* RNAi male flies. Approximately 3- to 5-day-old male offspring were used in experiments.

To reintroduce *bub1* into hemocytes or the whole body of *bub1^c04512^* flies, *bub1^c04512^*;*he-gal4* and *bub1^c04512^*;*da-gal4* flies were crossed with *bub1^c04512^*;*UAS-egfp-bub1* flies, respectively. Approximately 3- to 5-day-old male offspring were used in experiments.

### Cells and cells assays.

Drosophila S2*cells were cultured at 28°C in Schneider's Drosophila medium (Sigma) supplemented with 10% heat-inactivated fetal bovine serum (FBS) (Invitrogen). Vero cells and 293T cells were cultured at 37°C in Dulbecco's modified Eagle's medium (DMEM) (Invitrogen) supplemented with 10% heat-inactivated FBS.

To knock down *bub1 in vitro*, 8 × 10^6^ S2* cells were transfected with 15 μg dsRNA in a 6-well plate by using Lipofectamine 2000 (Thermo), and transfected cells were incubated at 28°C for 3 days before the following assay.

To knock down *bub1* in a human cell line, 293T cells were transfected with small interfering RNA (siRNA) (Ribobio Guangzhou) in a 6-well plate by using Lipofectamine 3000 (Thermo), and transfected cells were incubated at 37°C for 3 days before the following assay.

For virus replication and release analyses, the same numbers of dsRNA-treated cells were infected with DCV at an MOI of 0.01 in a 48-well plate at 28°C for 1 h and replaced with fresh medium, DCV RNA was extracted from S2* cells by TRIzol LS for replication analysis, and the supernatant was collected for a CPE assay for release analysis at the indicated times.

For virus binding assays, the same numbers of dsRNA-treated cells were infected by DCV or VSV at an MOI of 100 at 4°C (infection medium was supplemented with cycloheximide). After 30 min of infection, cells were collected and washed twice with prechilled phosphate-buffered saline (PBS) to remove unbound virus, and total RNA was extracted by using TRIzol.

For virus entry assays, the same numbers of dsRNA-treated cells were infected by DCV or VSV-GFP at an MOI of 100 at 28°C (infection medium was supplemented with cycloheximide). After 15 min or 30 min of infection, cells were collected and washed twice with prechilled PBS to remove unbound virus, and cells were then incubated with preheated trypsin at 37°C for the removal of virus that had already bound the cell membrane; after 10 min of incubation, cells were centrifuged and washed with PBS twice; and when only intracellular virus remained, total RNA was extracted by using TRIzol.

For dextran intake assays, the same numbers of dsRNA-treated cells were washed with serum-free S2 medium and treated with 25 μl 2.5-fold-diluted fluorescein-labeled dextran (70 kDa; Sigma) in a 96-well plate and incubated for 10 min at 28°C (protected from light), and cells were then washed with cold PBS and fixed with 4% paraformaldehyde (PFA) at 4°C for 30 min (protected from light). After fixation, cells were washed and plated on glass slides (protected from light) for observation by using a Zeiss LSM700 confocal microscope.

For Bub1 location analysis, S2* cells were transfected with pAGW-Bub1 for 72 h. After cells were counted, cells were divided into two parts and plated on a concanavalin A (ConA) (Sigma)-treated confocal dish, and the cells were allowed to attach for 2 h. One plate was infected by DCV at an MOI of 100 for 1 h (28°C), and the control group remained untreated. After infection, cells were fixed with 4% PFA at 4°C for 30 min and washed three times before observation by confocal microscopy.

For Bub1 pattern analysis, once large puncta of Bub1 (diameter of ≥1 μm) were formed in a cell, we defined the cell as having a “big puncta” phenotype. Otherwise, we defined cells as having a “small dots” phenotype if more than 5 small dots (<1 μm) appeared. The other cells were defined as having a “smear” phenotype.

### Cell proliferation and viability assays.

For cell proliferation assays, 8 × 10^6^ S2* cells were transfected with *rfp* dsRNA or *bub1* dsRNA in a 6-well plate for 3 days, and cell numbers were then counted. Cells were plated in a 96-well plate at a density of 2 × 10^5^ cells/well. Cell proliferation was assayed by using a CCK-8 kit (Dojindo) every day. Data for each time point represent results from 6 replicates.

For cell viability assays, 8 × 10^6^ S2* cells were transfected with *rfp* dsRNA or *bub1* dsRNA in a 6-well plate for 3 days. After that, cells were counted, and the same number of cells were plated in a 96-well plate at a density of 2 × 10^5^ cells/well every day. Cell viability was assayed by using a CCK-8 kit every day. Data for each time point represent results from 6 replicates.

### Constructs and double-strand RNAs.

pAGW-Bub1 and pAFW-AP-1-2-beta were constructed with the Gateway system (Invitrogen). *Bub1* and *ap-1-2-beta* full-length cDNAs were amplified from LD22858 and *w^1118^* genomic cDNAs, respectively, by using the primers listed in Table S1 in the supplemental material and then subcloned into the pENTR TOPO cloning vector (Invitrogen). These pENTR vectors were subsequently recombined into FLAG- or GFP-tagged destination vectors (Carnegie Institute for Science) by using LR Clonase (Invitrogen).

dsRNA was constructed by using an Ambion MEGAscript kit (Thermo) and stored at −20°C.

The sequences used are listed in Table S1 in the supplemental material.

### Fly infection and survival curves.

Newly emerged male adult flies were collected and maintained for ∼2 to 3 days on fresh fly food before infection. During infection, flies were anesthetized with CO_2_ and injected with pathogens by using a Drummond Nanoject II system. A total of 50.6 nl DCV (100 PFU per fly) or VSV (10^4^ PFU per fly) was injected into the thorax, and infected flies were maintained at 25°C. For bacterial infection, 50.6 nl Listeria bacteria (OD_600_ = 0.01) was injected into the anterior abdomen on the ventrolateral surface, and infected flies were maintained at 29°C. Twenty infected flies per vial were transferred to fresh food daily, and the number of dead flies was counted. Kaplan-Meier survival curves were generated, and statistical analysis was done using log-rank analysis by using Prism7 software. Survival was tested for each pathogen at least three times. For the screen data, the z-score of the median survival time was calculated by using Excel 2016.

### Virus and bacteria.

DCV was amplified in the S2* cell line. After 3 days of infection, culture medium was collected and centrifuged at 4°C for 10 min (10,000 × *g*), the supernatant was collected, and aliquots were frozen at −80°C. For VSV-GFP amplification, the Vero cell line was used. Culture medium was collected at 24 h postinfection and centrifuged at 4°C for 10 min (10,000 × *g*). The supernatant was collected and centrifuged at 68,000 × *g* for 1 h to concentrate the virus. Generally, 100 ml virus solution was condensed to 1 ml in Tris-HCl buffer (pH 7.2).

Listeria monocytogenes was cultured in brain heart infusion (BHI) medium until the OD_600_ reached ∼0.6 to 0.8. Bacteria were collected by centrifugation at 6,000 × *g* for 15 min, washed twice with sterile PBS, and resuspended in sterile PBS to an OD_600_ of 0.01.

### PFU and CFU counts.

For DCV titer determination, a CPE assay was used. S2* cells were plated into a 96-well plate at a density of 3,000 cells per well, and serially diluted virus was then added to the plate. After 3 days of incubation at 28°C, CPE was analyzed by microscopy, and titers were calculated.

For VSV-GFP titer determination, Vero cells were cultured in a 6-well plate until they reached 80% confluence. Culture medium was removed, and cells were washed with PBS. One milliliter of serially diluted virus was added, and cells were incubated at 37°C with constant shaking. After 1 h of infection, virus was removed, cells were washed twice with PBS, and 2 ml sterile agar–DMEM (40°C) was then gently added on the cell surface. After 24 h of incubation at 37°C, plaques were analyzed by microscopy, and titers were calculated.

For CFU assays of Listeria monocytogenes, flies were ground with 0.5-mm beads and serially diluted with LB medium. Samples were plated on an LB agar plate. For the gentamicin chase experiments, flies were injected with 50.6 nl of 1 mg/ml gentamicin or buffer 3 h prior to homogenization and plating.

### Wolbachia-free fly generation and detection.

Flies were cultured on fresh food with 200 μl 50 μl/ml tetracycline for three generations. cDNA was then extracted from 5 flies for each sample. Primers used for the detection of Wolbachia are listed in Table S1 in the supplemental material.

### Coimmunoprecipitation and Western blotting.

To test the interaction of Bub1 and AP-1-2-beta, S2* cells (8 × 10^6^) were cotransfected with 2.5 μg pAGW-Bub1and pAFW-AP-1-2-beta. Cells were cultured for 72 h and then incubated with or without DCV for 1 h (MOI = 100) at 28°C, cells were collected, and coimmunoprecipitation was carried out exactly as described previously (51), except that anti-FLAG (catalogue number ab290; Abcam) was used for immunoprecipitation and anti-GFP (M2; Sigma) antibody was used for immunoblotting. To test the interaction of Bub1 and the DCV capsid polyprotein, S2* cells (4 × 10^6^) were transfected with 1.25 μg pAGW or pAGW-Bub1. Cells were cultured for 72 h and then incubated with DCV for 1 h (MOI = 100) at 28°C. Cells were collected, coimmunoprecipitation was carried out; anti-GFP (catalogue number ab1218; Abcam) was used for immunoprecipitation, and anti-GFP (catalogue number ab290; Abcam) and anti-DCV (catalogue number ab92954; Abcam) antibodies were used for immunoblotting. The predicated molecular weights of the DCV capsid polyprotein were 100 kDa (full length) and several fragments of approximately 30 kDa (cleaved by proteases after infection) (6).

### Bub1 kinase inhibition.

Cells were pretreated with 10 μM or 40 μM 2OH-BNPP1 (catalogue number HY-102081; MCE) for 6 h, and cells were then infected with DCV (MOI = 0.01) for 12 h.

### Pastrel genotyping.

Fly DNA was extracted as mentioned above, and PCR assays were performed for pastrel genotyping. A total of 100 ng DNA was used as the template, the 512C primer was used to detect the R allele, the 512T primer was used to detect the S allele, and the *T_m_* (melting temperature) gradients were 54°C, 54.7°C, 55.5°C, 58°C, 59.7°C, 62.2°C, 63.7°C, and 64°C.

### Quantitative RT-PCR.

Total RNA was extracted with TRIzol or TRIzol LS (Invitrogen), and total RNA was then quantitated by using a NanoDrop 2000 instrument. The cDNA was prepared by reverse transcription with reverse transcriptase (Abm) with oligo(dT) or random primers. Abm Sybr green master mix (with Rox) and an ABI7500 system (Applied Biosystems) were used for quantitative RT-PCR. Each reaction was carried out in triplicate, and the copy numbers of the indicated genes were normalized to the value for endogenous ribosomal protein *rp49* mRNA. Oligonucleotide primers used are listed in Table S1 in the supplemental material.

### Statistical analysis.

All measurement data are expressed as means ± standard errors (SE). Comparisons of two samples were made using Student's *t* test, and comparisons of multiple samples were done by analysis of variance (ANOVA). Survival curves were compared using the Kaplan-Meier test. *P* values of less than 0.05 were considered statistically significant. All statistical analyses were performed with GraphPad Prism 7 software.

### Data availability.

The data that support the findings of this study are available from the corresponding authors upon request.

## Supplementary Material

Supplemental file 1
