# Supplementary material for: Bub1 Facilitates Virus Entry through Endocytosis in a Model of Drosophila Pathogenesis
Source: J Virol. 2018 Aug 29;92(18):e00254-18. doi: 10.1128/JVI.00254-18 (PMC6146689; doi:10.1128/JVI.00254-18)

SUPPLEMENTAL INFORMATION

Supplemental Information includes six figures, and one table.

Supplementary Figure

Figure S1 | Screen data

A, Fly lines used in second-round genetic screen. Z-score was calculated as mentioned in Methods and the dash lines indicate the significant interval (most left: susceptible section; most right: resistance section).

Figure S2 | Related to Figure 1 *bub1* deficient flies become resistant to DCV infection

A, *bub1* mRNA expression was restored in flies with p-element precise deletion. The change of expression was measured by qRT-PCR and normalized to that of w^{1118}.

B, Flies were treated by tetracycline for three generations and *Wolbachia* were detected by primers of *Wolbachia* specific *16s rRNA* and *wsp* gene. Wild type Or^R fly with *Wolbachia* was used as positive control.

C, *pastrel* genotyping by PCR. The R allele of *pastrel* was detected by 512C primers; The S allele of *pastrel* was detected by 512T primers. Tm gradients were a: 54℃, b: 54.7°C, c: 55.5°C, d: 58°C, e: 59.7°C, f: 62.2°C, g: 63.7°C, h: 64°C.

D, *bub1* knockdown efficiency in the whole body of flies measured by qRT-PCR.

All error bars represent standard error (s.e.) of at least three independent tests; n>15 flies for each group. *P<0.05, **P<0.01, ***P<0.001, ns, not significant. student's t-test (d), or one-way ANOVA (a).

Figure S3 | Related to Figure 3 *bub1* deficiency reduces DCV entry.

A, *bub1* knockdown efficiency in S2* cells, measured by qRT-PCR. The change of expression was normalized to that of *rfp* dsRNA treated cells.

B, *bub1* knockdown in S2* cells did not affect cell proliferation. Absorbance of OD_{450} was normalized to S2* cells treated with *gfp* dsRNA at day one after 3 days post *bub1*dsRNA transfection.

C, *bub1* knockdown in S2* cells did not affect cell viability. Absorbance of OD_{450} was normalized to S2* cells treated with *gfp* dsRNA at each day, respectively.

Characterization of DCV infection kinetics in *Drosophila* S2* cells (D, E). D, DCV binding kinetic at different MOI. The solid line showed the linear regression, the dashed lines represents 95% confidence band of the best-fit line. r^2=0.9862, p<0.0001. E, DCV entry kinetic at different time points with MOI=100. Solid line, non-linear regression was used, r^2=0.9567. Dashed line, linear regression was used, r^2=0.9998, p=0.008.

All error bars represent standard error (s.e.) of at least three independent tests; *P<0.05, **P<0.01, ***P<0.001, ns, not significant. student's t-test(a), two-way ANOVA (b, c).

Figure S4 | Related to Figure 4 Bub1 is involved in regulation of endocytosis.

A, Endocytosis component-deficient flies were resistance to DCV infection compared to wild-type (w^{1118}) flies. Heterozygote flies were obtained by crossing with w^{1118} virgin.
B, *bub1* mRNA expression of the whole body before or after DCV infection, measured by qRT-PCR and normalized to the wild type control.
C, *bub1* overexpression in S2* cells had no effect on DCV entry ability (30min post infection). DCV entry ability indicated by DCV genome RNA level in S2* cells quantified by qRT-PCR and normalized to vehicle plasmid transfected cells.
D, DCV genome level of indicated cells measured by qRT-PCR at 12h post infection, normalized to that of control.
All error bars represent standard error (s.e.) of at least three independent tests; n>60 flies (a) for each line or n>15 (b) for each time point. *P<0.05, **P<0.01, ***P<0.001, ns, not significant. Kaplan–Meier (a), or student's t-test (b, c, d).

Figure S5 | Survival rates of flies after VSV infection.
A, Survival rates of wild-type (w^{1118}) flies and $bub1^{c04512}$ mutant flies post VSV-GFP injection.
All error bars represent standard error (s.e.) of at least three independent tests; n>60 flies (a). *P<0.05, **P<0.01, ***P<0.001, ns, not significant. Kaplan–Meier (a).

Figure S6 | 2OH-BNPP1 affects DCV replication.
A, Inhibition of Bub1 kinase activity by high level of 2OH-BNPP1 reduced DCV loads. DCV RNA level of indicated cells measured by qRT-PCR at the indicated time and was normalized to that of DMSO treated cells.
B, 40uM 2OH-BNPP1 treatment had no impact on Bub1/AP-1-2-beta interaction upon DCV infection.
All error bars represent standard error (s.e.) of at least three independent tests; *P<0.05, **P<0.01, ***P<0.001, ns, not significant. two-way ANOVA (a).

Supplementary Table1 primers and RNAi used in experiments

Rp49-F AGATCGTGAAGAAGCGCACCAAG
Rp49-R CACCAGGAACTTCTTGAATCCGG
Bub1-F TGTGATCAGGTGCTGAAACG
Bub1-R TTAGACTAGCGTTTGGGGCG
VSV-G-F TGCCCCACTGTCCATAACTC
VSV-G-R CAGCCATCTCGAACCAGACA

DCV-F TCATCGGTATGCACATTGCT
DCV-R CGCATAACCATGCTCTTCTG
GFP-F GAGCTGAAGGGCATCGACTT
GFP-R TTCTGCTTGTCGGCCATGAT
Vago-F TGCAACTCTGGGAGGATAGC
Vago-R AATTGCCCTGCGTCAGTTT
Vir-1-F GATCCCAATTTTCCCATCAA
Vir-1-R GATTACAGCTGGGTGCACAA
pAGW-bub1-F CACCATGGCCATGCACTCGTACATG
pAGW-bub1-R TCGTCGATGCAGGATGTTGGA
AP-1-2-beta-F: CACCATGACTGATTCCAAGTACTTC
AP-1-2-beta-R: TTA TGGAGAACGGATGATGGC
FIL-F TGAGGAAGATAATGACGG
FIL-RCCTCTATCCTCTTTCAACC
Wsp-F CATTGGTGTTGGTGTTGGTG;
Wsp-R ACCGAAATAACGAGCTCCAG
dsBub1-F TTAATACGACTCACTATAGGGAGAGGGCACGCACCAAAGTTAAG
dsBub1-R TTAATACGACTCACTATAGGGAGAATGGCCTCCTTGTCCATGTG
dsGFP-F TTAATACGACTCACTATAGGGAGAGGCAAGCTGACCCTGAAGTT
dsGFP-R TTAATACGACTCACTATAGGGAGACTCAGGTAGTGGTTGTCGGG
Bub1 siRNA 1 CCAGAATGGCAGTGTATTA
Bub1 siRNA 2 CAACAACAATACAGGTTAT

Bub1 siRNA 3 GCCTCATGCTGAAGAGTTT
512C-F CAGCATGGTGTCCATGAAGTC
512T-F CAGCATGGTGTCCATGAAGTT
512-R ACGTGATCAATGCTGAAAGT

Supplemental Figure 1

A

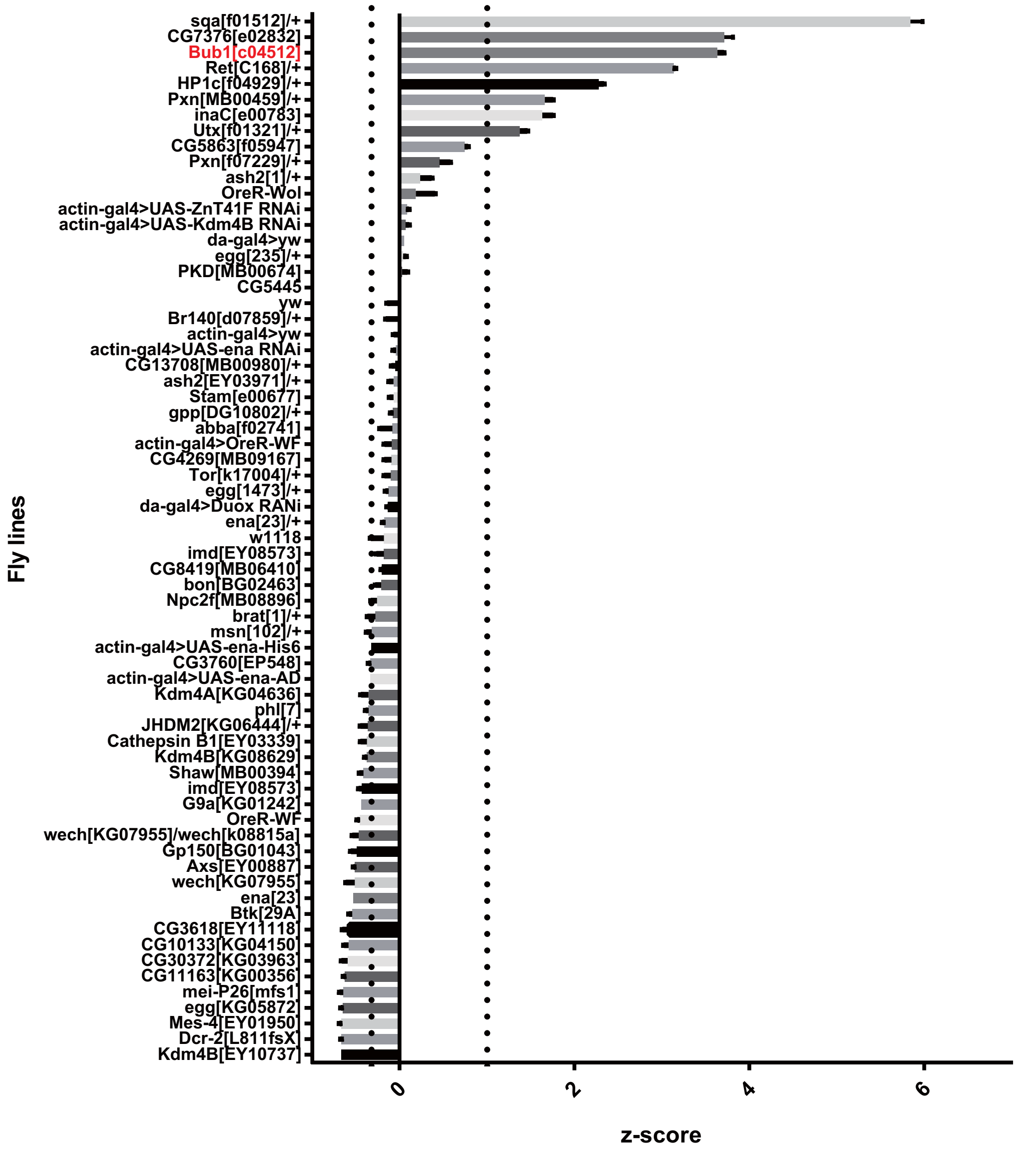

Supplemental Figure 2

A

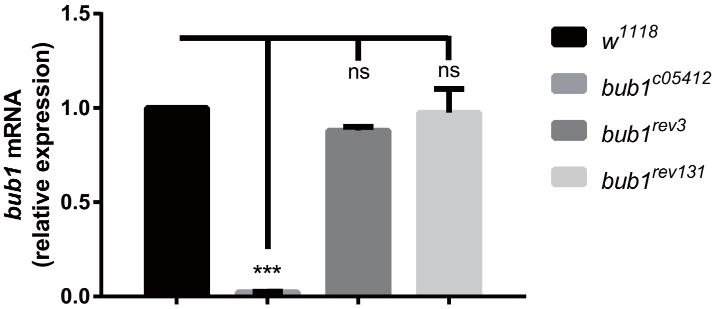

B

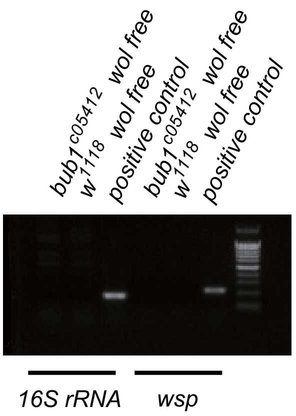

C

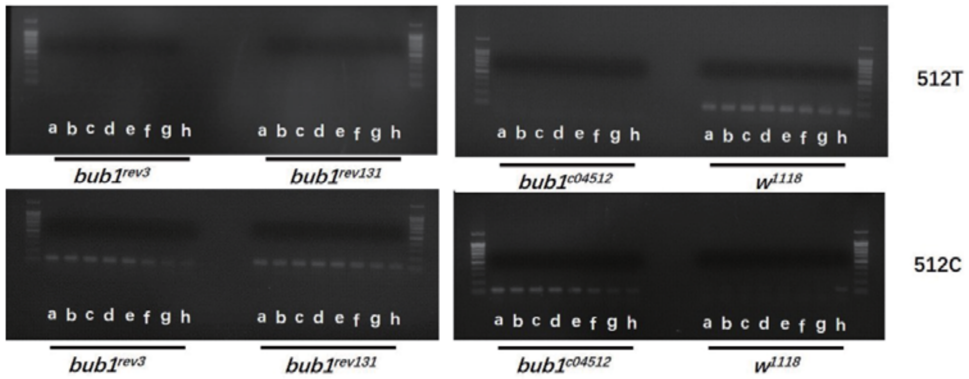

D

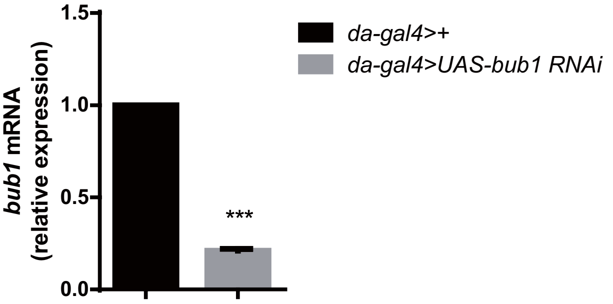

Supplemental Figure 3

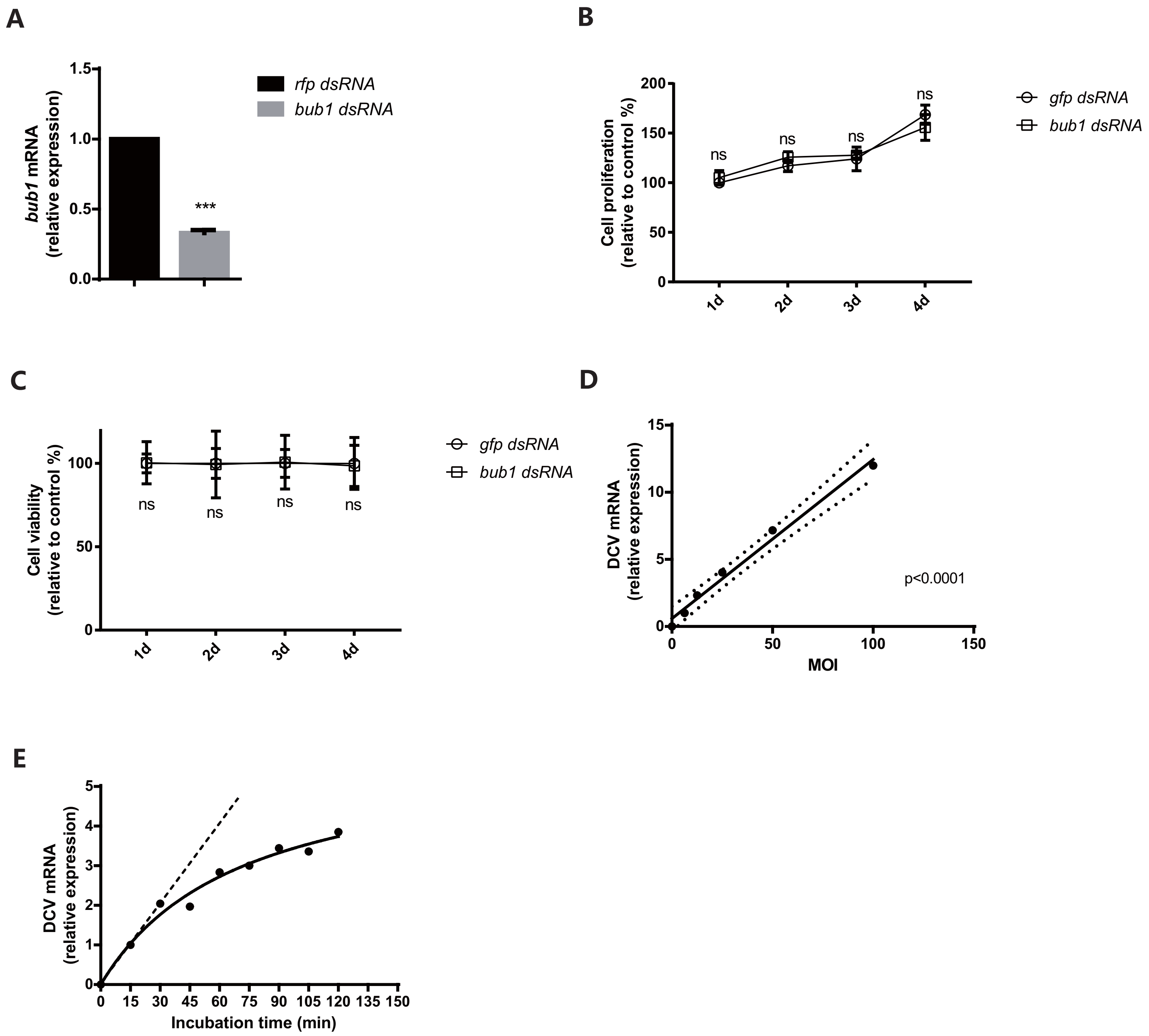

Supplemental Figure 4

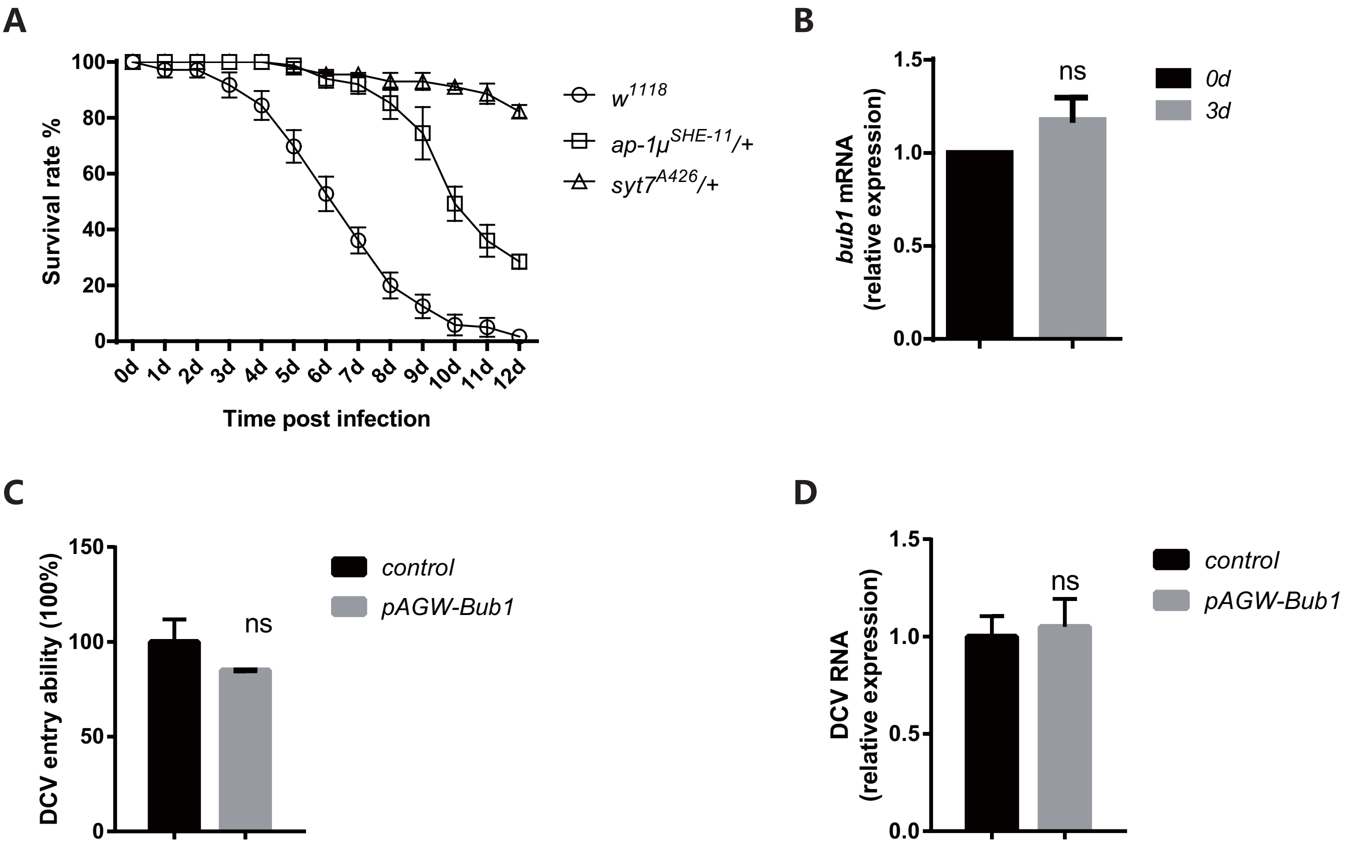

Supplemental Figure 5

A

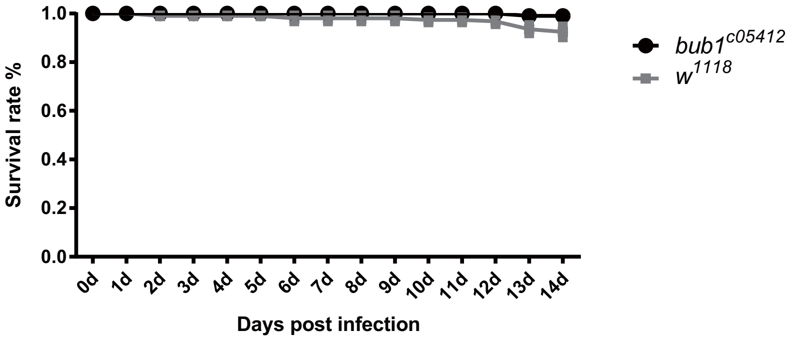

Supplemental Figure 6

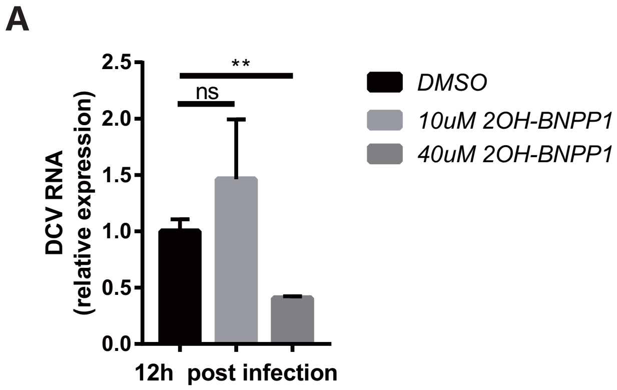

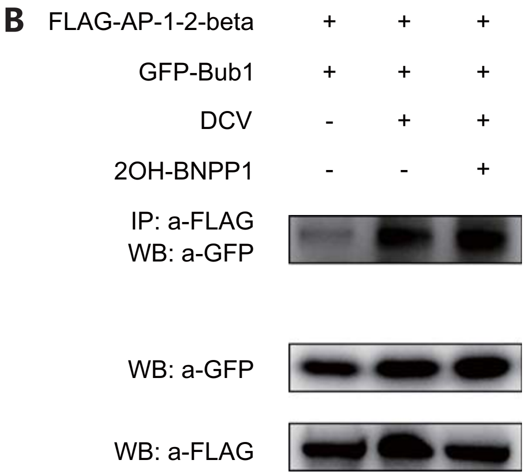

Supplement: Supplemental file 1 [file zjv018183851s1.pdf]
